# Australian general practitioner perceptions of the detection and screening of at-risk drinking, and the role of the AUDIT-C: a qualitative study

**DOI:** 10.1186/1471-2296-14-121

**Published:** 2013-08-20

**Authors:** Chun Wah Michael Tam, Nicholas Zwar, Roslyn Markham

**Affiliations:** 1 School of Public Health and Community Medicine, The University of New South Wales, Level 3 Samuels Building, UNSW Sydney, NSW 2052, Australia; 2 The New South Wales Institute of Psychiatry, North Parramatta, NSW, Australia

**Keywords:** Alcohol drinking, Primary health care, General practitioners, Substance abuse detection, Health knowledge, Attitudes, Practice

## Abstract

**Background:**

At-risk drinking is common in Australia. Validated screening tools such as the AUDIT-C have been promoted to general practitioners (GPs), but appear rarely used and detection of at-risk drinking in primary care remains low. We sought to describe Australian GP perceptions of the detection and screening of at-risk drinking; to understand their low uptake of alcohol screening questionnaires, and in particular, their attitude to the adoption of the AUDIT-C.

**Methods:**

Semi-structured focus group interviews of four groups of GPs and GP trainees were conducted in metropolitan Sydney between August and October 2011. Audio recordings were transcribed and analysed using grounded theory methodology.

**Results:**

We identified four main themes: there was consensus that detecting at-risk drinking is important but difficult to do, social and cultural attitudes to alcohol consumption affect willingness to ask questions about its use, the dynamics of patient-doctor interactions are important, and alcohol screening questionnaires lack practical utility. Analysis suggests that the conceptual barriers to detecting at-risk drinking were: community stigma and stereotypes of “problem drinking”, GP perceptions of unreliable patient alcohol use histories, and the perceived threat to the patient-doctor relationship.

**Conclusion:**

This small exploratory study found that the practice of, and barriers to, detecting at-risk drinking appear to be inextricably linked to the sociocultural beliefs surrounding alcohol use. Screening questionnaires such as the AUDIT-C are not designed to address these issues. In the current context, it is unlikely that approaches that focus on the use of these tools will be effective at improving detection of at-risk drinking by GPs.

## Background

“At-risk drinking” refers to the consumption of alcohol in such a manner that an individual is placed at increased risk of alcohol-related harm [1]. This phenomenon is common in Australia – approximately 2 in 10 adults in the general population [2] , and 3 in 10 adults attending general practice are at-risk drinkers [3].

GPs are seen to have a major role in reducing these harms – they are often an individual’s first point of contact with the health system and have good access to the at-risk population [4]. Furthermore, brief alcohol interventions delivered in primary care are believed to be effective in assisting patients reduce their alcohol consumption [5].

However, at-risk drinkers must first be identified for brief alcohol interventions to be offered. There is evidence dating back some years that Australian GPs did not identify the majority of these patients [6,7]. Recent evidence suggests that the under-detection of at-risk drinking remains an enduring phenomenon internationally [8,9] and that brief interventions are rarely offered [3,9,10].

Alcohol screening questionnaires have been widely promoted in the academic literature and clinical practice guidelines as a means to address the problem of under-detection [5,11-13]. Australian GPs have been encouraged to routinely screen patients for at-risk drinking [11,14,15] using the Alcohol Use Disorders Identification Test (AUDIT) [16], or the shorter AUDIT-C [17]. Despite the enthusiasm for these tools in the literature and guidelines, they do not appear to have been embraced by GPs. Uptake of alcohol screening questionnaires is low [18,19].

There has been some qualitative analysis of the barriers to using alcohol screening questionnaires in primary care, but not in the Australian general practice setting [20]. In this exploratory qualitative study we describe the beliefs and attitudes expressed by a number of Australian GPs and GP trainees on the detection of, and screening for, at-risk drinking with a focus on the AUDIT-C, the questionnaire recommended in Australian guidelines [11]. We aimed to understand the reasons for the low uptake of alcohol screening questionnaires, and the overall low detection of at-risk drinking in the Australian general practice context.

## Methods

We used Straussian grounded theory, a qualitative research method that is exploratory in nature, and seeks to generate theory from analysis of data [21]. We chose this method over other qualitative methods as it is well suited to examining the underlying social processes of specific phenomena, and was consistent with our constructivist ontological perspective – that knowledge can be built from the perspectives of our participants [22]. Its elements include: (i) coding of collected data into categories, (ii) an iterative process whereby earlier analyses inform further data collection, (iii) the use of a constant comparison technique in the analytic process to discover the conceptual interactions between categories, and (iv) the development of theory that emerges from, and is grounded in, the data [21].

### Focus groups – the practices and participants

Group interactions in focus group interviews can provide rich data of individual and shared beliefs and attitudes. Moreover, GPs in focus group interviews have been found to express common shared narratives as a group of medical professionals, in which all participants were able to contribute [23]. Given the relatively small scale of our study, we opted to use this research technique rather than individual interviews. Our focus group interviews were designed to be similar to the routine clinical meetings of the practices so that the participants would be in a familiar environment. They were conducted in a meeting room within each practice, and the participants of each focus group were GPs of that practice.

A convenience sample of GP teaching practices in Sydney affiliated with either the University of New South Wales, or GP Synergy Ltd. (a provider of GP vocational training) was approached to participate in the study. These practices were chosen as: (i) each included a GP with teaching or research interests who could act as a contact, and (ii) had a sufficient number of GPs that focus group interviews could be run. The planned size of each focus group was 4–8 participants.

A total of nine group practices were approached and four agreed to participate. A number of barriers were experienced in recruitment. Some practices had an insufficient number of interested participants and finding a mutually convenient time of sufficient length (45 to 60 minutes) for the doctors to meet proved challenging. We planned to approach additional practices to conduct further focus group interviews if theoretical saturation had not been reached with the four groups, but this turned out to be unnecessary. The focus groups were held between August and October 2011. The groups consisted of GP supervisors, other GPs, and GP registrars, with a total number of 19 participants. Table 1 lists the practice and focus group characteristics, and Table 2 describes participant characteristics.

**Table 1:** Characteristics of the participating practices and focus groups

	**No. participants**	**No. doctors in the practice**
**Practice 1**	6	10
**Practice 2**	7	9
**Practice 3**	4	6
**Practice 4**	2	7

All four practices were self-described as “well established”, “appointment based”, “urban”, and used “Best Practice Clinical” software for clinical records.

**Table 2:** Summary of participant characteristics (self-reported)

	**Female**	**Male**	**Age (yr) mean (range)**	**GP experience (yr) mean (range)**
**GP supervisors**	5	2	51 (37–73)	22 (11–36)
**GP registrars**	1	3	35 (30–40)	3 (1–6)
**Other GPs**	3	4	46 (31–72)	14 (5–26)
**Other **^**†**^	1		25	n/a
**Total**	10	9		
**Other details**	4/19 doctors had worked in a drug and alcohol unit.			
	16/19 doctors were Australian medical graduates.			
	Average hours worked per week: 32			
	Average patients seen per week: 83			
	Average appointment length: 16.4 min			

^†^ One doctor was a hospital-based prevocational trainee on a primary care rotation.

All focus group interviews were moderated by the first author (CT). A semi-structured approach was employed with five questions used as triggers if the issues did not arise naturally in discussion (Table 3). The participants were given a printed copy the AUDIT-C questionnaire [24] towards the end of the focus group interviews to stimulate discussion on this tool. Each focus group was digitally recorded, transcribed, and de-identified.

**Table 3:** Focus group trigger questions


**1**	What proportion of adult patients (aged over 18 years) presenting to Australian general practice do you think drink at at-risk levels?
**2**	What do you think about identifying patients with at-risk/problem drinking in primary care?
**3**	How do you currently identify these patients?
**4**	What do you think about routine alcohol screening?
**5**	What do you think about the AUDIT-C tool?

### Data analysis

The transcripts were coded by analysing the data line by line, and initial interpretations made. Important issues and themes that were identified from the earliest focus groups were then explored more deeply in subsequent ones. For instance, GP perceptions of the influence of sociocultural attitudes on alcohol use emerged strongly in the initial focus groups and were not entirely expected. GP beliefs, attitudes and experiences on how these sociological factors influenced patient behaviour, clinician behaviour and the patient-clinician dynamic were sought explicitly in the latter focus groups.

Throughout the process the team met at regular intervals to discuss interpretations of the data, though the coding was predominantly performed by CT. Discrepancies in interpretation were discussed in these meetings until consensus was reached. He monitored coding consistency by continually reviewing and comparing the coding categories and sub-categories. The relationship between dominant themes and the development of theory was assisted with the use of diagrams and visualising the data with tree-maps. Analysis of the transcripts, codes, categories and themes were performed using QSR International Nvivo 9 software.

### Ethics approval

This study received approval from the University of NSW Medical and Community Human Research Ethics Advisory Panel, and the NSW Institute of Psychiatry Human Research Ethics Committee.

## Results

### Themes

Our analysis identified four major themes that help explain the low rates of detection of at-risk drinking in Australian primary care, and the lack of use of alcohol screening questionnaires by GPs. The following sequence reflects the conversational flow of the group interviews. The GPs typically started with dialogue on their beliefs and attitudes on detecting at-risk drinking. They reflected on and debated the wider role of societal attitudes and sociocultural contexts, as well as focussing on the issues at the level of the consultation. Lastly, they specifically discussed their views on screening when they were presented with the printed copy of the AUDIT-C.

#### Detecting at-risk drinking is important but difficult to do

Focus groups began with each participant asked to estimate the proportion of Australian adults presenting to GPs who were at-risk drinkers. The average of the estimates was 31%. Leading on from the initial discussion of prevalence, almost all GPs agreed that the detection of at-risk drinking was important:

…And I think it’s certainly important … as … doctors taking care of the Australian population. That’s [alcohol use] certainly … one of the things we should be trying to identify and educate our patients about and hopefully be able to engage them in modifying their behaviour when they’re willing… (D18)

Most GPs perceived that the accuracy of the assessment was crucial:

…But I also think people will say, “I only drink … I don’t drink very often,” but that could be two or three big nights on the weekend which we think is excessive drinking. So I think it’s really important to try … to clarify for them exactly what they do… (D8)

There was a general view that alcohol use histories from patients were unreliable and typically under-estimated. This was perceived as a major barrier to detection of at-risk drinking:

…I think people are extraordinarily resourceful in the ways in which they’re able to potentially cover up behaviour they don’t want others to see. So, people are likely to under-report the amount that they’re drinking, either knowingly or subconsciously… (D18)

Even when GPs identified a patient with at-risk drinking, they seemed reluctant to label it in clinical records. Some elaborated that at-risk drinking was not a diagnosis or disease, so shouldn’t be labelled. Others were concerned with issues of patient confidentiality and the potential for the labels to be passed on to third parties, e.g., to insurers. Many GPs were concerned of being perceived as judgemental by patients. Nevertheless, there was a broad agreement that alcohol use is conceptually within the scope of health issues that can be addressed in the GP setting.

Some GPs expressed their sense of ineffectiveness in promoting change in their patients’ alcohol use behaviours:

…I’m not that bad at detecting it. I’m bad at doing anything about it. As in … I honestly don’t know sometimes if I find out the answer whether it makes that much of a difference of what they end up, or what I end up doing… (D11)

#### Impact of social and cultural attitudes

Sociocultural attitudes and their impacts emerged strongly as a theme in all groups. It was widely perceived that some patterns of drinking that were risky were “normal” and even expected in many Australian community contexts:

…it’s very socially acceptable to drink… It’s completely normal for a teenager to come out of school at the age of 17 and start, you know, having binge drinks with his mates at bars and clubs… For girls to be given free drinks by bouncers and … bartenders at nightclubs… (D16)

These GPs believed that at-risk drinking labels were perceived to be shameful by the community, and so individuals reject these labels for their personal drinking patterns:

It’s socially unacceptable to say you’re a heavy drinker, but it’s actually socially acceptable to be a heavy drinker. That’s the problem… (D11)

Several GPs compared smoking to drinking to highlight the impact of community beliefs:

…And it’s very few people who deny smoking is a bad thing. Even quite heavy smokers… you know… there’s hardly any that just think you’re making it up. Whereas, I think with alcohol it’s seen to have benefits… (D5)

In their discussions, the GPs described three main impacts on patient beliefs and behaviour arising from these sociocultural attitudes: (i) some patients were defensive about or hide their use of alcohol, (ii) others did not recognise the health risks from drinking, and (iii) drinking guidelines were not always perceived as “reasonable”:

…Because then everybody who saw themselves as a perfectly reasonable person without an alcohol problem, if you drank that much [referring to National Health and Medical Research Council guidelines]… they became a binge drinker, and I think that people… weren’t interested to… they didn’t listen any more. They just felt that was such an unreasonable label that they didn’t want to listen to what it might be based on. (D8)

#### Dynamics of patient-doctor interactions

Many GPs indicated that asking patients about their alcohol use was a potential threat to the patient-doctor relationship. At times, they were unwilling to introduce this topic into the consultation, being self-conscious about being perceived as moralising by the patient. The situation most frequently identified as challenging was a presenting problem that was seen as unrelated to alcohol use:

…And someone with a cold, unless they’re a new patient … it wouldn’t be something [alcohol use] that you’d necessarily ask about… And they might be a bit affronted if you did, if you couldn’t figure a way of bringing it in. (D6)

However, assessment of alcohol use was perceived as rather less threatening to the relationship when the patient was new to the practice, when it was part of an explicit “health check”, or when completing a computer health record:

…I’ve taken to the habit of updating the smoking and the alcohol history in the last year… and they’re quite happy to be asked it, especially when you say, “Look, I’m just doing a refresh of your file [electronic record] and updating of all the information”. (D12)

Furthermore, there seemed to be presentations that actively triggered assessment of alcohol use. This was described to be the case for certain blood test abnormalities (elevated gamma-GT) and mental illness by many GPs:

…It often comes up in mental health consults, if they’re depressed … it’s almost like an open door to ask a little about alcohol then. (D3)

Several GPs identified other patient presentations that facilitated assessment, such as hypertension, traumatic accidents, and pregnancy and fertility.

#### Alcohol screening questionnaires lack practical utility

The GPs framed their discussions on alcohol screening tools by considering the issues of practicality and utility. Most GPs knew of the CAGE questionnaire [25], and a few were aware of the AUDIT and AUDIT-C. The universal experience, however, was that they either rarely or never used these tools. Those who had experience using these questionnaires did not use them for the purpose of routine screening. Rather, it had either been in a practice research setting, or they used the questions as a framework for exploring alcohol use with patients they had already identified with an alcohol use disorder:

…Yeah, I suppose the times I’ve done it [CAGE] is just… actually getting the patient to engage with why I think they’ve got an alcohol problem. (D12)

When the GPs were given the AUDIT-C questionnaire, the overwhelming and uniform perception was that it would over-identify patients with at-risk drinking. The question items were considered reasonable, but the scoring was met with marked scepticism.

…it’s [detecting] a lot of people that probably are not really having a problem… it seems silly… (D17)

Several GPs elaborated that although they believed that screening for at-risk drinking was good in theory, it needed to be performed in all patients to be useful:

…But, as for existentially, as a thing, yes, I think it would be excellent if we knew everyone’s alcohol status. It would be a good thing to aspire to. (D5)

…you could be fooled, so it’s better… it’s… a population screen really isn’t it? (D7)

However, the pragmatic consensus was that routine screening with questionnaires was too difficult to implement. The GPs felt that they could not, and would not perform it consistently in practice.

### Barriers to the detection of at-risk drinking

In synthesising the codes, concepts and themes, sociocultural factors appeared to have a key influence on the willingness of GPs to attempt to identify at-risk drinking by patients in their clinical practice. Our analysis suggests that this results from three interconnected attitudes and beliefs: (i) community stigma and stereotypes of “problem” drinking, (ii) GP perceptions of unreliable patient alcohol use histories, and (iii) the perceived threat to the patient-doctor relationship from alcohol use assessment.

The first barrier, community stigma and stereotypes of “problem” drinking, might have important direct and indirect effects on the detection of at-risk drinking. The GPs were concerned about the possibility of being seen as passing moral judgement on their patients. This might explain their reluctance to assess alcohol consumption when they perceived it as irrelevant to the presenting problem, as well as their reluctance to label drinking as “at-risk”.

The second barrier, GP perceptions of the unreliability of patient alcohol use histories, can be seen as interacting with, and perhaps, even as a consequence of the first. In response to the stigma of being labelled a “problem drinker”, patients may portray themselves as “normal” drinkers, regardless of their use of alcohol. This could explain the GPs’ beliefs and observations that alcohol use histories were not only unreliable but also under-estimates.

The perception of the potential threat to the patient-doctor relationship is the third barrier. The GPs indicated that they might opt to preserve the therapeutic alliance by strategically avoiding questions that could lead to conflict – especially if they were pessimistic of the likelihood of engendering patient behaviour change. This perception may inhibit GPs from inquiring and clarifying patient alcohol use in some clinical situations.

## Discussion

Many of the concepts and themes that emerged in our study were consistent with those identified in qualitative research of GPs in different times and places [20,26-29]. For instance, some GPs were previously noted to have been concerned about stigmatising alcohol users and being seen as “moralists” [26,27,29]. GPs have been observed to refrain from challenging patients regarding their alcohol use to preserve relationships [30]. The general unreliability of patient histories has also been described as a barrier to detection before [29,31]. Similarly, some researchers have previously expressed that probing patient usage of alcohol could be perceived as an attack on the patient’s integrity [27].

It is uncontroversial that there are large cross-cultural variations in social attitudes to alcohol [32]. Comparisons between countries suggest that alcohol-related problems are not directly related to consumption levels, but rather, are associated with “specific cultural factors, relating to beliefs, attitudes, norms and expectancies about drinking” (p 6) [32]. Australia has been described as having a “Temperance” or “ambivalent” drinking culture, and alcohol in Australia is associated with violence and anti-social behaviour (p 6), use of celebration as an “excuse” for drinking (p 9), and drinking places more likely to be enclosed, insular, or secretive (p 26) [32]. In a recent cross-sectional survey, alcohol was perceived by Australians as the drug of most serious concern to the community [2].

Although the broader social influence on detection of at-risk drinking has been previously identified [26,28], this emerges particularly strongly in our study. We postulate that the practice of attempting to detect at-risk drinking by GPs is also inextricably linked to the drinking culture in the broader society. In essence, the beliefs and attitudes of individual patients, doctors, and the expectations of medical consultations, can be seen as sitting within sociocultural contexts and being shaped by them [33].

Moreover, we propose that the significance of these factors on the detection of at-risk drinking in primary care has been substantially underemphasised – recent categorisations of barriers have focussed mainly on clinician factors (e.g., knowledge, time, confidence), some organisational factors (e.g., lack of financial incentives) and occasionally patient factors [20].

There are two important implications of the current study. Firstly, it needs to be noted that alcohol screening questionnaires were not designed to address sociological issues. If the enduring barriers to GPs identifying at-risk drinkers mainly arise as a consequence of such factors, then this could largely explain the low uptake of tools like the AUDIT-C in routine practice. GPs are not likely to use or recommend a tool that they do not perceive as useful.

Secondly, strategies to improve GP detection of at-risk drinking have often been focussed on overcoming perceived deficiencies in clinician factors, e.g., education, training, and support for using alcohol screening questionnaires and brief interventions. If the aforementioned societal level factors are more relevant, then these strategies are unlikely to be effective on their own. There is some evidence that this is the case [34,35].

### Limitations and strengths of this study

This was an exploratory qualitative study designed to develop hypotheses and understand why alcohol screening tools that have the potential to be effective in identifying patients with at-risk drinking are not used in primary care. The most important weakness of the study is that it sampled only the views of only a small number of Australian doctors in GP teaching practices in metropolitan Sydney. GPs in teaching practices who participate in alcohol research are likely to be more knowledgeable and confident in the detection and management of at-risk drinking, and may not be representative of GPs in general. Moreover, recruiting in teaching practices might have led to groups being relatively homogenous in their views – resulting in premature theoretical saturation of themes.

As the GPs in each practice were well known to each other there was a friendly atmosphere in the focus groups interviews, which facilitated participant interaction and discussion. However, this social dynamic may have also led the participants into displaying socially desirable attitudes, as well as viewpoints that are consistent with past interactions with their colleagues. It is conceivable also that the participants who were trainees might not have voiced opinions that contradicted those of their supervisors, or more senior colleagues.

Nevertheless, despite some of these limitations, the similarity of the identified themes to previous qualitative research in this field is a strength of this study and supports the applicability of the results [20,26,28]. In addition, this study adds contemporary Australian GP perspectives, which has been largely absent in the published literature. Lastly, it offers an interpretation that may explain the low uptake of alcohol screening questionnaires by GPs, at least in practices similar to those included in this study.

### Direction of future research

The significance of sociocultural factors on the detection of at-risk drinking that emerges from our data was hypothesised three decades ago, “in the long run, medical intervention is influenced by extraneous political and social factors” (page 405) [26], and “responsibility for the failure of early diagnosis and treatment of alcohol misuse in medical settings lies neither with the patient or physician but with the relationship of their roles” (page 427) [36]. This hypothesis deserves further exploration and testing.

Furthermore, although alcohol screening questionnaires are designed to be used in primary care, there is an absence of evidence that GPs find the routine use of these tools acceptable. GPs who have been involved in implementing alcohol screening in their practices have typically reported critical and negative experiences [31,34]. As there is little evidence that the recommendations promoting GP use of screening questionnaires have made a meaningful impact on routine clinical practice [19,34,35], future research and recommendations in this field need to be inclusive of GP perspectives.

## Conclusions

The detection of at-risk drinking in Australian primary care appears to be heavily influenced by sociocultural factors. These shape the conceptual barriers to detection – the stigma of alcohol dependence, GP perceptions of unreliable patient alcohol use histories, and the threat to patient-doctor relationships from alcohol use assessment. Screening questionnaires do not appear to address these barriers.

Although detecting at-risk drinking was considered important, alcohol screening questionnaires were not perceived as part of routine practice by our participants. Universal screening was seen as impractical, and the AUDIT-C in particular was considered to have poor practical utility. In the current context, it seems unlikely that approaches that focus on GP use of these tools will be successful at improving detection of at-risk drinking in primary care.

## Competing interests

The authors declare that they have no competing interests.

## Authors’ contributions

CT conceived and designed the study, conducted the data collection, analysis, and drafted the manuscript under the mentorship and guidance of NZ and RM. All authors had full access to the data. All authors contributed to the latter drafts, and approved the final version.

## Pre-publication history

The pre-publication history for this paper can be accessed here:

http://www.biomedcentral.com/1471-2296/14/121/prepub
